# Neural Substrates for Early Data Reduction in Fast Vision: A Psychophysical Investigation

**DOI:** 10.3390/brainsci14080753

**Published:** 2024-07-26

**Authors:** Serena Castellotti, Maria Michela Del Viva

**Affiliations:** 1 Department of Translational Research on New Technologies in Medicine and Surgery, University of Pisa, 56126 Pisa, Italy; 2 Department of Neurosciences, Psychology, Drug Research and Child Health (NEUROFARBA), University of Florence, 50135 Florence, Italy; maria.delviva@unifi.it

**Keywords:** fast vision, visual data reduction, early feature extraction, constrained maximum entropy, visual sketches, visual saliency, psychophysics, flicker adaptation, contrast sensitivity, magnocellular pathway

## Abstract

To ensure survival, the visual system must rapidly extract the most important elements from a large stream of information. This necessity clashes with the computational limitations of the human brain, so a strong early data reduction is required to efficiently process information in fast vision. A theoretical early vision model, recently developed to preserve maximum information using minimal computational resources, allows efficient image data reduction by extracting simplified *sketches* containing only optimally informative, salient features. Here, we investigate the neural substrates of this mechanism for optimal encoding of information, possibly located in early visual structures. We adopted a flicker adaptation paradigm, which has been demonstrated to specifically impair the contrast sensitivity of the magnocellular pathway. We compared flicker-induced contrast threshold changes in three different tasks. The results indicate that, after adapting to a uniform flickering field, thresholds for image discrimination using briefly presented sketches increase. Similar threshold elevations occur for motion discrimination, a task typically targeting the magnocellular system. Instead, contrast thresholds for orientation discrimination, a task typically targeting the parvocellular system, do not change with flicker adaptation. The computation performed by this early data reduction mechanism seems thus consistent with magnocellular processing.

## 1. Introduction

At any given moment, the visual system must process a huge amount of information coming from the external world to rapidly identify the most relevant elements for initiating adaptive behaviors [1]. This necessity clashes with the brain’s physical limitations on processing visual information [2]. The creation of an accurate representation of the visual scene in the shortest possible time also demands a significant amount of energy [3,4], mainly due to the high rate of neuronal spikes [5]. All this emphasizes the existence of an early visual information bottleneck [6], operating a strong data reduction [7,8]. It has been hypothesized that early visual mechanisms perform such compression by extracting visual *sketches* based on a limited number of primitives (edges and bars) [9,10], as those found in primary visual areas [11], which will be analyzed by further levels of processing.

Here, we consider a recent model of early feature extraction which explicitly aims at reducing information through the selection of visually salient features [12]. Unlike other early vision models [13,14,15], the proposed model is not based on the known physiological properties of the visual system; rather, it is based only on general principles such as the amount of information transmitted to the next levels of processing and the physical constraints of a biological system [12]. First, the model assumes that, at an initial phase of the visual stream, the extensive input data stream is reduced by selectively filtering only those elements of the input that correspond to a predefined set of features, ignoring all other information (*pattern matching*). Second, the model assumes a finite number of visual features that the system can detect in the input (*limited capacity*). Third, there is a strict upper limit on the total amount of data that can be generated as output for transmission to subsequent processing stages (*fixed output bandwidth*). Lastly, the system is designed to transmit the maximum possible information to the next stages of processing within the system’s constraints (*maximum entropy output).* The output of this process is the extraction of a few pieces of information (*optimal* features) which preserve the maximum amount of information while minimizing computational costs. The proposed model thus assumes that the visual system uses only these features to build early compressed representations of visual scenes (sketches) to be transmitted to further levels of analysis [12].

When applied to binarized images, the constrained maximum-entropy model outlined above leads in fact to the extraction of simplified but very informative sketches, obtained by filtering images only with *optimal* features (small image patches) [12]. Psychophysical tests showed that these sketches allow the same performance in image discrimination as their unfiltered originals, suggesting that, in fast vision, it is sufficient to just a few optimally informative details to discriminate images [12]. In more recent studies, we also demonstrated that *optimal* features are recognized as more visually salient and strongly attract attention and eye movement compared to *non-optimal* features [16,17,18,19].

All these results indicate that highly informative local features are prioritized during rapid image processing, thereby forming a *bottom-up saliency map* at an early stage of visual processing. Such findings align with studies proposing that these maps are represented in early sensory cortices and require fast computation [20,21,22].

The present work employs psychophysical methods to unveil the specific neural pathways responsible for the efficient encoding of information, by exploring the relative contribution to this mechanism of the magnocellular (MC) and parvocellular (PC) systems [23,24].

### 1.1. Spatiotemporal Characteristics of MC and PC Systems

These two main parallel pathways originate in the primates’ retina, where there are two major classes of ganglion cells, P- and M-cells, with distinct characteristics. P-cells have a slow and sustained response and are most sensitive to chromatic stimuli of high spatial and low temporal frequencies, while M-cells have a rapid, transient response preferring achromatic stimuli of low spatial and high temporal frequencies [25,26,27,28,29,30,31]. This clear division of functions has been however challenged by electrophysiology, showing significant spatial frequency-overlap sensitivity between the MC and PC systems, especially before MT level [32,33,34,35,36]. The two pathways travel from the retina to the lateral geniculate nucleus (LGN) and maintain their anatomical separation until they arrive at the visual cortex [23,24]. Overall, the magnocellular system seems to be crucial for contrast and movement analysis, while the parvocellular system appears to be involved in the analysis of the details and color perception [37,38]. Then, visual information is processed predominantly via two main pathways: the ventral pathway, which extends toward the temporal lobe and is involved in objects’ recognition, and the dorsal pathway, which travels to the parietal lobe (and subsequently to the frontal lobes) and conveys information about the objects’ location [39].

The characteristics of the parvocellular and magnocellular cells have been classically investigated by neurophysiology studies [23,24,25]. Furthermore, some studies demonstrated how it is possible to use simple behavioral tasks to selectively target these two neural systems and measure their properties without adopting invasive techniques. Pokorny and Smith’s research group began a series of psychophysical experiments to separately measure achromatic contrast discrimination mediated by the MC and PC pathways, through the well-known *pedestal* paradigms [40]; for a review see [41]. Additionally, neurophysiological studies showed that prolonged exposure to high-contrast stimuli causes significant suppression of the contrast response in ganglion and LGN cells of the MC system but not in those of the PC system [42]. In parallel, many psychophysical studies showed that contrast adaptation over time reduces contrast sensitivity [43,44]. However, these studies have not explored the localization of this effect, ignoring whether it is caused by changes occurring in the neural responses of the MC and/or PC systems. To delve deeper into this aspect, a variation of the steady- and pulsed-pedestal paradigms has been developed introducing temporal adaptation to a 50% contrast square-wave modulated luminance *flicker* [45]. It was found that, when observers are adapted to the flicker before a steady-pedestal task, contrast thresholds for discrimination in the task increase. Thus, it has been concluded that a prolonged adaptation to a flicker allows for selective desensitization of the magnocellular system [45]. Also, in line with neurophysiology studies claiming that the MC system is more sensitive to high temporal frequency [42,43], they demonstrated that a 10 Hz flicker caused a stronger lowering of contrast sensitivity compared to that obtained with a 2 Hz flicker [45].

### 1.2. Research Rationale

Coming back to the objective of the current study, a recent study becomes particularly relevant [46]. Here, the reference model [12] was employed to investigate the potential role of fine-scale, information-rich color features in the context of competition among different types of information within a limited-capacity resource system. They concluded that, due to the pressure to optimize limited resources, the human visual system adheres to the maximum-entropy principle, prioritizing luminance information for rapid image discrimination over other potential information sources such as color. This finding suggests that the early system in charge of optimal encoding of information may be insensitive to color information. This is the case of the magnocellular pathway, which is also known to have high light/dark contrast detection and short-latency responses [27,29,47,48], which makes it suitable to build a reliable percept in a very short time, but this deserves further investigation.

Therefore, our prediction is that fast and efficient visual feature extraction, based on constrained maximum-entropy criteria, is carried out by the magnocellular system. To this purpose, we exploit a flicker adaptation paradigm, as the one designed in [45], which allows the selective desensitization of the magnocellular channel.

Our experiment engages participants in three tasks, each including a baseline and a flicker-adaptation condition. In the main experimental task, observers have to discriminate a target image from a distractor (Two-Alternative Forced-Choice procedure—2AFC) based on the brief presentation of a corresponding sketch, extracted according to the reference model. The performance is measured as a function of the contrast level of the sketches with and without the flicker adapter. If flicker adaptation significantly increases the contrast threshold for perception of sketches, by analogy with steady-pedestal paradigms [45], we can infer that the magnocellular system is the one in charge of optimal encoding of information in fast vision. A motion discrimination task (control task I) is introduced to compare flicker-induced effects on a task typically attributed to the magnocellular system in the same experimental conditions (same adapter, stimuli durations, luminance, etc.). This choice is based on the evidence that selective deactivation of primates’ LGN magnocellular layers causes a dramatic drop of contrast sensitivity for motion direction discrimination in the corresponding visual field region [49]. An orientation discrimination task (control task II) is also introduced to confirm that our adapter does not increase contrast thresholds in a task typically attributed to the parvocellular system.

The outcome of this study will then shed light on the anatomical substrates of the proposed early compressive mechanism leveraging on a non-invasive, simple behavioral technique.

## 2. Materials and Methods

### 2.1. Participants

Thirty young adults (10 males and 20 females; mean age = 25.2 ± 2.6 years) participated in the experiment. All observers had normal or corrected-to-normal visual acuity and no history of visual or neurological disorders. All participants were naive as to the purpose of the study and gave written informed consent before participation. The study was conducted according to the guidelines of the Declaration of Helsinki and approved by the local ethics committee (“Commissione per l’Etica della Ricerca”, University of Florence, 7 July 2020, n. 111).

### 2.2. Experimental Set-Up

All stimuli were programmed on an ACER computer running Windows 7 and displayed on a gamma-corrected CRT Silicon Graphics monitor (frame rate = 120 Hz, resolution = 1280 × 960 pixels, luminosity level = 99, contrast level = 54). The observer was seated 57 cm away from the monitor (38.5° × 29.5°) with a chin rest to ensure head stability. Stimuli were presented, and data were collected using programs developed with the Psychophysics Toolbox extensions [50,51,52] for Matlab (R2016b version; The MathWorks Inc., Natick, MA, USA). Luminance levels of the stimuli were measured using a Konica Minolta CS 100-A spectroradiometer (Konica Minolta Sensing Americas, Inc., Ramsey, NJ, USA). All experiments took place in a dark room, and participants provided manual responses using a standard Dell keyboard.

### 2.3. Procedures and Stimuli

The experiment is composed of three different tasks: an image discrimination task (experimental task), a motion discrimination task (control task I), and an orientation discrimination task (control task II). Each task included a baseline and a flicker-adaptation condition, performed two hours apart. Tasks were completed in a random order across participants over three different non-consecutive days. Each participant undertook a total of 6400 trials. In all tasks, the screen background was grey (pixel value: 127; 12 cd/m^2^). For each task, we tested stimuli with different contrast levels (detailed below), with a brief pause every 100 trials. The contrast values were selected based on some pilot trials, which led us to use very different contrast levels and step sizes for the three tasks to achieve performances ranging approximately from 50% to 100% and properly detect 80% thresholds. However, the upper limit was never reached for the experimental task, as we will show and discuss in the following sections. Pilot trials also allowed us to determine the breaks necessary to avoid attentional loss and eye strain.

**Figure 1:**
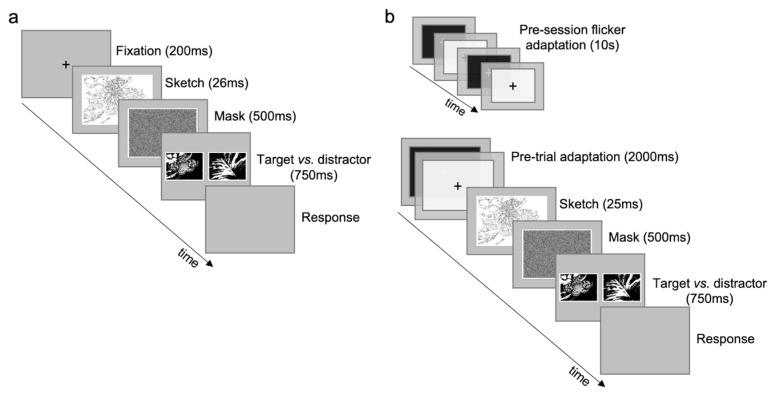
Image discrimination (experimental task). (**a**) Baseline condition. (**b**) Flicker-adaptation condition. Stimuli are not depicted with original dimensions to ease illustration. Examples with 100% contrast sketches and images are shown; see Figure S1a for a faithful depiction of the entire range of contrasts.

The experimental task consisted of a 2AFC match-to-sample procedure with backward masking (Figure 1). In each trial, a sketch was presented in the center of the screen (20° × 17°) for 25 ms, followed by a masking image (17° × 14°) for 500 ms, and then two images (17° × 14°) were shown side-by-side for 750 ms (separated from 1° visual angle). One of the images was the unfiltered version corresponding to the sketch (target), while the other was a distractor; both images were randomly chosen from the dataset. Sizes and durations of stimuli are comparable to those used in [12]. Observers were asked to indicate the correct match between the sketch and the image, reporting the response with a computer key (e.g., left arrow for the image on the left). As soon as the participants gave the response, the next trial started. At any given trial, the images and the mask stimulus had the same contrast as the sketch, which could randomly have one of the following values: 0.02, 0.05, 0.07, 0.1, 0.15, 0.2, 0.3, 0.5, 0.7, 1 (Figure S1a). In the baseline condition before each trial, a central fixation cross was presented for 200 ms (Figure 1a); in the adaptation condition, a flicker adapter was shown at the beginning of the session for 10 s and before each trial for 2 s (Figure 1b). During the adaptation, a cross was always visible in the center of the screen, and observers were instructed to maintain the fixation. The experimental task consisted of 2000 trials in total, 1000 trials for each condition (100 for each contrast). Images (768 × 576 pixels, obtained from [53]) were converted to 1-bit luminance (black/white). Sketches were derived from these images (pixel values: black = 0, white = 255; luminance values: black = 7 cd/m², white = 22 cd/m²) by retaining only the features identified as *optimal* by the reference constrained maximum-entropy model (by using as model parameters N = 50 and W = 0.05, as in [12]) and blanking all other parts. All possible 3 × 3 pixel patches, centered on every pixel of the image and including overlaps, were considered [12]. The mask was a random pixel image. The flicker-adapter stimulus, used to reduce contrast sensitivity, consisted of two uniform fields with same size and position of the sketch, alternating at 10 Hz as in [45] (50% Michelson contrast, luminance white 22 cd/m^2^, luminance black 7 cd/m^2^).

**Figure 2:**
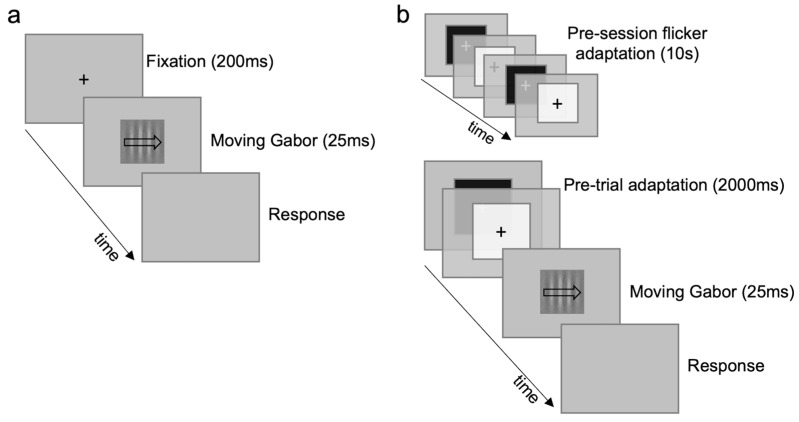
Motion discrimination (control task I). (**a**) Baseline condition. (**b**) Flicker-adaptation condition. Gabors are not depicted with original dimensions and contrast to ease illustration; see Figure S1b for a faithful depiction of the entire range of contrasts.

Control task I consisted of a motion direction discrimination task (Figure 2), in which one moving Gabor (5°) was presented in the center of the screen for 25 ms. Participants were required to discriminate the direction of motion (e.g., left arrow for leftward motion). The Gabor patch was a vertical sinusoidal grating (1 c/deg) embedded in static gaussian noise (σ = 50), moving rightward or leftward at 10 Hz temporal frequency. The spatial frequency value corresponds to the peak of the contrast sensitivity for gratings moving at 10 Hz [54]. Temporal frequency was the same as in [45]. Gabor motion direction changed randomly in each trial. Twelve Gabor contrasts were tested: 0.01, 0.02, 0.03, 0.04, 0.05, 0.06, 0.07, 0.08, 0.09, 0.1, 0.15, 0.2 (Figure S1b)—randomly chosen in each trial. The task included one baseline (Figure 2a) and one flicker-adaptation condition (Figure 2b). Adaptation periods and adapter parameters were the same as in the experimental task, but the flicker field size matched that of the Gabor patch. Control task I consisted of a total of 2400 trials, 1200 trials for each condition (100 for each contrast).

**Figure 3:**
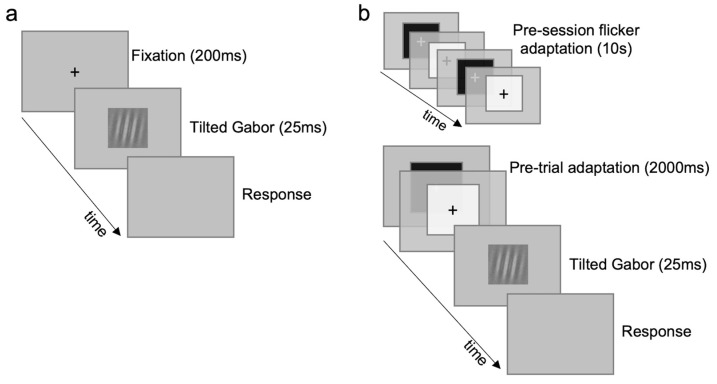
Orientation discrimination (control task II). (**a**) Baseline condition. (**b**) Flicker-adaptation condition. Gabors are not depicted with original dimensions and contrast to ease illustration; see Figure S1c for a faithful depiction of the entire range of contrasts.

Control task II consisted of an orientation discrimination task (Figure 3), in which one tilted Gabor (5°) was presented in the center of the screen for 25 ms. Participants were required to discriminate the Gabor orientation (e.g., right arrow for clockwise orientation). The Gabor patch was a sinusoidal grating (5 c/deg) embedded in static gaussian noise (σ = 50), whose orientation changed randomly in each trial (±10° with respect to vertical). The spatial frequency value corresponds to the peak of the contrast sensitivity for static gratings [54]. Ten contrast levels were tested: 0.01, 0.02, 0.03, 0.04, 0.05, 0.06, 0.07, 0.08, 0.09, 0.1 (Figure S1c)—randomly chosen in each trial. One baseline (Figure 3a) and one flicker-adaptation condition (Figure 3b) were tested. Adaptation periods and adapter parameters were the same as in the control task I. An example of a trial for each condition is reported in Figure 3. Control task II consisted of 2000 trials in total, 1000 trials for each condition (100 for each contrast).

### 2.4. Data Processing and Statistical Analyses

For each participant in each task, we calculated the probability of correct responses as a function of the stimulus contrast in the baseline and the flicker-adaptation condition. Data were fitted with cumulative Gaussian error functions, and contrast thresholds for discrimination (*T*), defined as the contrast value yielding 75% of correct responses, were extracted. Since threshold distributions revealed deviations from normality (Shapiro–Wilks tests yield *p* < 0.05), non-parametric statistical tests were used. Specifically, contrast thresholds in the baseline vs. flicker-adaptation condition of each task were compared with paired-sample Wilcoxon signed-rank tests. The effect sizes of the differences between conditions were estimated by rank-biserial correlation coefficient (rrb) with 95% confidence intervals. Finally, relative percentage changes in the threshold due to flicker adaptation in different tasks were calculated, taking into account the different maximum contrasts of each task, using the following formula:

$$\mathrm{Threshold}\;\mathrm{relative}\;\mathrm{percentage}\;\mathrm{change}=\left(\frac{T_{Flicker}-T_{Baseline}}{Max\;contrast}\right)\times 100$$

Non-parametric one-way repeated-measures, Friedman’s test, with Conover post hoc comparisons (Bonferroni correction), were then used to compare differences between threshold elevations in the three tasks. To compute the effect sizes of these differences, Kendall’s W value was used.

## 3. Results

**Figure 4:**
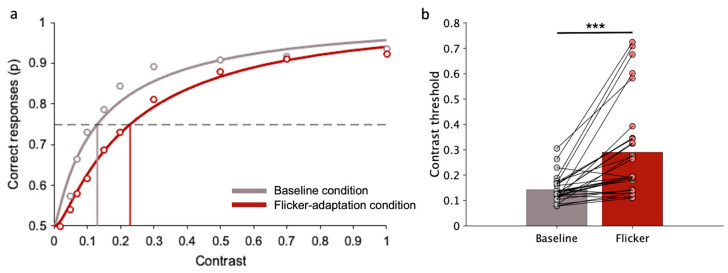
Image discrimination (experimental task): contrast threshold in baseline and flicker-adaptation condition. (**a**) Example of correct responses as a function of sketch contrast, obtained by pooling data across all participants. The points represent the proportion of times the image discrimination task was correctly completed at each contrast level. The curves represent cumulative Gaussian error fits of the data. The vertical lines indicate contrast thresholds, which correspond to the contrast values resulting in 75% correct responses (dashed line). (**b**) Bars represent the average contrast threshold across participants. Points correspond to the contrast threshold of each participant under that specific condition. Lines connect thresholds of the same participants in the two conditions. Asterisks mark statistically significant paired-sample Wilcoxon signed-rank tests (Bonferroni correction): *** *p* < 0.001.

Figure 4a shows the probability of correct responses in the image discrimination task as a function of sketch contrast, obtained by pooling together data from all participants (for illustration purposes only, statistical analysis has been carried out on individual data). For both conditions, at the lowest contrast (0.02) performance is at a chance level and then increases with sketch contrast; however, the performance is constantly lower when observers are presented with the flicker-adapter stimulus. Figure 4b shows group average and individual thresholds in the two conditions. Statistical analysis confirms that the average contrast threshold is significantly higher in the flicker-adaptation condition (M = 0.14, SD = 0.05) than in the baseline condition (M = 0.29, SD = 0.18; W(29) = 13, *p* < 0.001; rrb = −0.95, 95% CI [−0.97, −0.87]). The increase in contrast threshold due to flicker adaptation is present in all participants, except for a few cases (three out of 30 participants).

**Figure 5:**
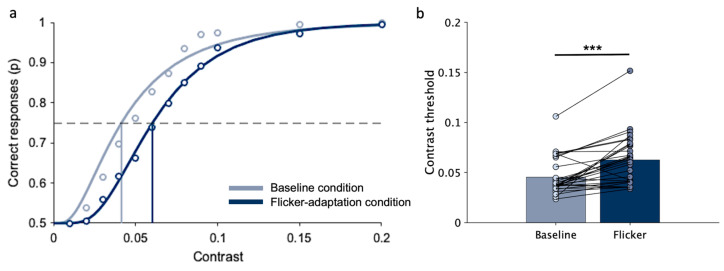
Motion discrimination task (control task I): contrast threshold in baseline and flicker-adaptation condition. (**a**) Example of correct responses as a function of Gabor contrast, obtained by pooling data across all participants. The points represent the proportion of times the motion discrimination task was correctly completed at each contrast level. The curves represent cumulative Gaussian error fits of the data. The vertical lines indicate contrast thresholds, which correspond to the contrast values resulting in 75% correct responses (dashed line). (**b**) Bars represent the average contrast threshold across participants. Points correspond to the contrast threshold of each participant under that specific condition. Lines connect thresholds of the same participants in the two conditions. Asterisks mark statistically significant paired-sample Wilcoxon signed-rank tests (Bonferroni correction): *** *p* < 0.001.

Figure 5a shows the probability of correct responses in the motion discrimination task as a function of the Gabor contrast, obtained by pooling together data from all participants. For both conditions, at the lowest contrast (0.01) performance is at a chance level and then increases with stimulus contrast reaching 100% of correct responses for the highest contrast (0.2). As for the image discrimination task, the performance is constantly lower when observers are presented with the flicker-adapter stimulus. Figure 5b shows group average and individual thresholds in the two conditions. Statistical analysis confirms that the average contrast threshold is significantly higher in the flicker-adaptation condition (M = 0.045, SD = 0.018) than in the baseline condition (M = 0.063, SD = 0.025; W(29) = 34, *p* < 0.001; rrb = −0.85, 95% CI [−0.93, −0.70]). The increase in contrast threshold due to flicker adaptation is present in all participants, except for a few cases (four out of 30 participants). 

**Figure 6:**
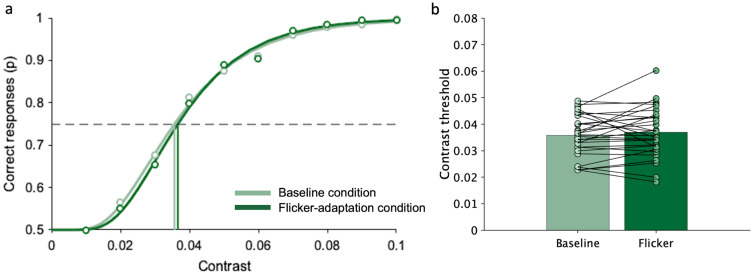
Orientation discrimination (control task II): contrast threshold in baseline and flicker-adaptation condition. (**a**) Example of correct responses as a function of Gabor contrast, obtained by pooling data across all participants. The points represent the proportion of times the orientation discrimination task was correctly completed at each contrast level. The curves represent cumulative Gaussian error fits of the data. The vertical lines indicate contrast thresholds, which correspond to the contrast values resulting in 75% correct responses (dashed line). (**b**) Bars represent the average contrast threshold across participants. Points correspond to the contrast threshold of each participant under that specific condition. Lines connect thresholds of the same participants in the two conditions.

Figure 6a shows the probability of correct responses in the orientation discrimination task as a function of the Gabor contrast, obtained by pooling together data from all participants. For both conditions, at the lowest contrast (0.01) performance is at a chance level and then increases with stimulus contrast reaching 100% of correct responses for the highest contrast (0.1). Differently from the other two tasks, the performance increases in the same way with and without the presence of the flicker-adapter stimulus. Figure 6b shows group average and individual thresholds in the two conditions. Statistical analysis confirms that the average contrast threshold is the same in the flicker-adaptation condition (M = 0.036, SD = 0.008) and in the baseline condition (M = 0.037, SD = 0.009; W(29) = 179, *p* = 0.28). 

**Figure 7:**
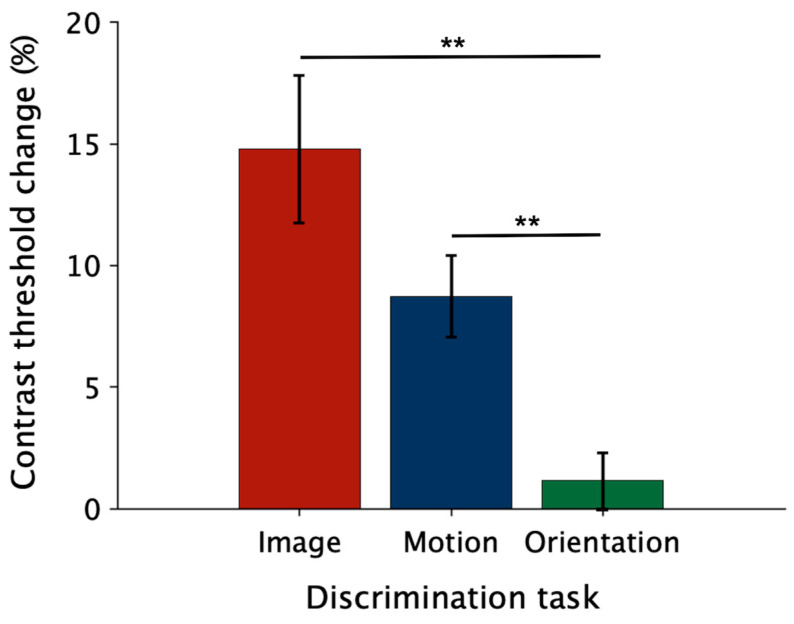
Flicker-induced threshold elevation for different tasks. Bars show average percentage threshold changes in the flicker-adaptation relative to the baseline condition for each task. Asterisks mark statistically significant Conover post hoc comparisons (Bonferroni correction): ** *p* < 0.01.

Figure 7 shows the average percentage threshold changes in the flicker-adaptation relative to the baseline condition for each task (see *Data processing and statistical analyses* section). The statistical analysis shows that contrast threshold flicker-induced changes differ across tasks (χ^2^(2) = 14.47, *p* < 0.001, W = 0.24). Specifically, there are significant differences between the (absent) threshold change in the orientation task and threshold elevation found in the image discrimination (*t*(58) = 3.2, *p* = 0.006) and motion discrimination tasks (*t*(58) = 3.36, *p* = 0.004). Note that threshold elevation is about 15% for image discrimination and about 8% for motion discrimination; however, these do not significantly differ (*t*(58) = 0.13, *p* > 0.05).

## 4. Discussion

The present study aims to investigate the relative contributions of the magnocellular (MC) and parvocellular (PC) pathways in early visual processes responsible for the efficient coding of information based on constrained maximum-entropy criteria [12].

The magnocellular system, highly sensitive to contrast and with very short response latencies [27,29,47,48], appears to be suitable for the extraction of informative features when the visual system is forced to rapidly and reliably process the maximum possible information, to allow the implementation of automatic behaviors. Our investigation then exploits a flicker adaptation paradigm, designed to selectively desensitize the magnocellular pathway [42,45], and assess its impact on various visual discrimination tasks.

The main experimental task revealed that flicker adaptation significantly increases the contrast threshold required for participants to correctly match a sketch to its corresponding unfiltered image. Given that the sketches used in the discrimination task were generated based on *optimal* features extracted under constrained maximum-entropy criteria, the increased difficulty in discriminating these sketches after flicker adaptation suggests that the magnocellular system is integral to this rapid and efficient visual processing.

Flicker adaptation also leads to a significant increase in the contrast threshold required for motion direction discrimination. This validates the effectiveness of our flicker adaptation paradigm in targeting tasks typically in charge of the magnocellular system. Thresholds for orientation discrimination are not enhanced by introducing the flicker adapter. This result aligns with our initial predictions, as orientation discrimination is primarily mediated by the parvocellular pathway, and therefore should not be affected by the flicker adaptation [42,45]. Our results are consistent with those obtained by exposing observers to a flicker adapter similar to the one used here, even though the experimental conditions and the paradigms (pulsed- and steady-pedestal paradigms) are completely different [45].

With this study, we may thus conclude that the MC pathway, known to be dedicated to motion analysis [37,38], may be the neural substrate in charge of the perception of optimally informative visual features. Salient sketches contain only these high-frequency spatial features, which require the highest computational effort for their processing given their prevalence in visual scenes. This observation seems in contrast with the role of the magnocellular system in sketch analysis, as the MC neurons are generally considered mainly sensitive to low rather than high spatial frequencies [25,26,27,28,29]. However, an existing body of electrophysiological evidence showed sensitivity of MC cells to high frequencies too, with a significant overlap of frequency preference between the MC and PC systems, especially before MT level [32,33,34,35,36]. This is also supported by classic psychophysical studies suggesting the existence of two distinct motion channels: a “sustained” channel tuned to high-spatial and low-temporal frequencies and a “transient” channel tuned to low-spatial and high-temporal frequencies [55,56,57]. These authors argue that the temporal tuning of these channels not only serves to enhance motion sensitivity but also renders them an ideal mechanism for the analysis of the spatial structure of the target [56,57].

Our results also raise other considerations. When considering performance levels for target stimuli with different contrasts, our data show substantial differences across tasks. Particularly, observers’ performances with sketches never reached the top, nor with full contrast, with a maximum of ~90% for all participants. This is probably due to the intrinsic difficulty of the task, which involves very brief presentation times and similar images to discriminate. In other words, this specific task has an intrinsic error rate of about 10%. This is not true for the control tasks, where participants reach maximum performance for much lower contrast values. This probably depends on the fact that it is easier to make a left-right discrimination of basic features such as orientation and movement in a very short time, rather than a sketch-based image discrimination.

It is worth mentioning that the paradigm used in this study is somewhat innovative, given that we used a basic non-invasive psychophysical method to shed light on the spatiotemporal properties of the neural mechanisms involved in building a rapid bottom-up saliency map of the visual scene. Overall, the findings obtained in this work corroborate the specificity of the flicker adaptation effect on the magnocellular system and reinforce the conclusion that this simple paradigm may be a sensible technique to investigate neural substrates underlying different tasks.

The present work thus adds a piece to bridge the gap between the theoretical model of visual data compression and the empirical evidence of the neural pathways involved. Once the magnocellular pathway’s pivotal role in early visual processing is revealed, it remains to be understood at which stage of this stream the compressive mechanism is located. In other words, other techniques can be used in the future to identify which visual area constitutes the information bottleneck in charge of the generation of a “bottom-up saliency map”. Given the rapid response times of neurons in the primary visual cortex [58,59], the resemblance of V1 receptive fields with the spatial structure of the predicted *optimal* features [11,12], and the fact that V1 is the largest visual area with significant energy consumption and high neural input/output ratio [3,60], it is reasonable to assume that V1 is the most probable neural substrate for this process. Also, other studies have proposed that V1 creates a bottom-up saliency map enabling a ”lossy pre-attentive selection of information“ [20,61,62], like the one implemented by the reference model [12].

From an applied perspective, understanding the mechanisms of visual data compression and the roles of different visual pathways can inform the design of more effective artificial vision systems, such as in computer vision and artificial intelligence. For instance, algorithms that mimic the magnocellular system’s efficiency in feature extraction could enhance image recognition systems’ speed and accuracy.

Our findings may also have implications for models of visual attention, suggesting that the magnocellular pathway may play a critical role in guiding eye movements toward the most salient features to maximize information in natural visual scenes (e.g., [63]).

## 5. Conclusions

To conclude, we found that efficient feature extraction, as predicted by the constrained maximum-entropy model [12], seems to rely on the rapid and robust processing capabilities of the magnocellular pathway, known to be dedicated to motion analysis. These findings thus prompt possible extensions of the theoretical framework of the model. A future extension from the spatial to the spatio-temporal domain would be even more biologically plausible than the current implementation, taking into consideration the well-established role of *motion* as a fundamental property of visual saliency [64,65,66] in the early visual system [67,68,69,70].

## Data Availability

All data are available from the Zenodo database (https://doi.org/10.5281/zenodo.12913621).

## Supplementary Materials

The following supporting information can be downloaded at: https://www.mdpi.com/article/10.3390/brainsci14080753/s1, Supplementary Figure S1. Examples of stimuli showing the entire range of contrasts in different tasks. (a) Example of a sketch in the image discrimination task (experimental task). (b) Vertical Gabor used in the motion discrimination task (control task I). (c) Tilted Gabor (+10°) used in the orientation discrimination task (control task II).
