# Supplementary material for: Neural Substrates for Early Data Reduction in Fast Vision: A Psychophysical Investigation
Source: Brain Sci. 2024 Jul 26;14(8):753. doi: 10.3390/brainsci14080753 (PMC11352587; doi:10.3390/brainsci14080753)
Supplement: Supplementary file 1 [file brainsci-14-00753-s001.zip › brainsci-3103448-supplementary.pdf]

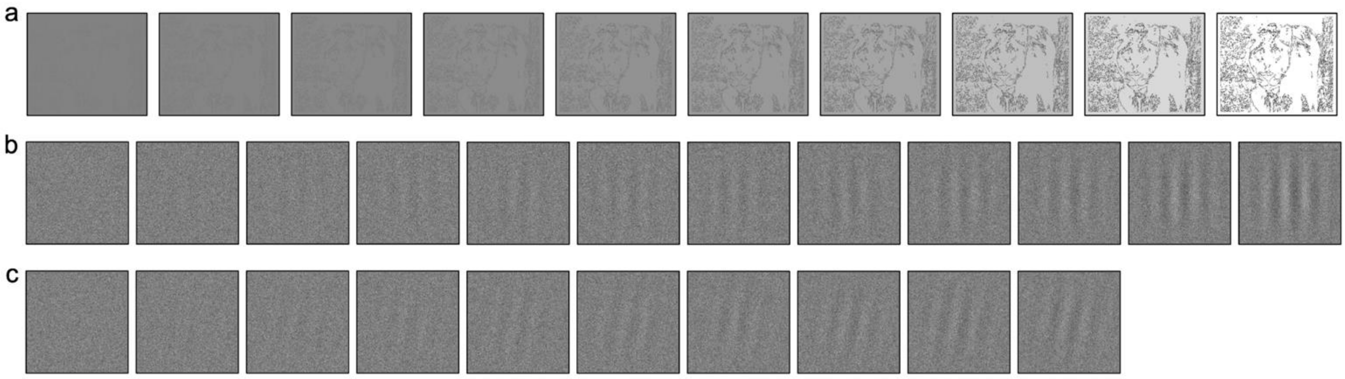


**Supplementary Figure S1. Examples of stimuli showing the entire range of contrasts in different tasks.** **(a)** Example of a sketch in the image discrimination task (experimental task). **(b)** Vertical gabor used in the motion discrimination task (control task I). **(c)** Tilted gabor (+10°) used in the orientation discrimination task (control task II).
